# Césarienne à Lubumbashi, République Démocratique du Congo I: fréquence, indications et mortalité maternelle et périnatale

**DOI:** 10.11604/pamj.2017.27.72.12147

**Published:** 2017-06-01

**Authors:** Xavier Kinenkinda, Olivier Mukuku, Faustin Chenge, Prosper Kakudji, Peter Banzulu, Jean-Baptiste Kakoma, Justin Kizonde

**Affiliations:** 1 Département de Gynécologie-Obstétrique, Faculté de Médecine, Université de Lubumbashi, République Démocratique du Congo; 2 Institut Supérieur des Techniques Médicales de Lubumbashi, République Démocratique du Congo; 3 Département de Gynécologie-Obstétrique, Faculté de Médecine, Université de Kinshasa, République Démocratique du Congo

**Keywords:** Césarienne, fréquence, indications, évolution, mortalité maternelle et périnatale, Lubumbashi, République Démocratique du Congo, Cesarean section, frequency, indications, change, maternal and perinatal mortality, Lubumbashi, Democratic Republic of Congo (DRC)

## Abstract

**Introduction:**

La fréquence de la césarienne (CS) ne cesse d'augmenter ces dernières décennies mais cette augmentation diffère énormément d'un pays à un autre et dans un même milieu, d'une institution médicale à une autre. L'objectif de cette étude est d'étudier la fréquence, les indications et la morbi-mortalité maternelle et périnatale de la césarienne (CS) à Lubumbashi, République Démocratique du Congo (RDC).

**Méthodes:**

Étude multicentrique, rétrospective, descriptive et analytique de 3643 CS consécutives sur un total de 34199 accouchements dans cinq formations hospitalières de référence à Lubumbashi (RDC) entre le 1er janvier 2009 et le 31 décembre 2013. Les données sociodémographiques, les indications, l'environnement obstétrical et la morbi-mortalité maternelle et périnatale ont été analysés au logiciel Epi Info 2011. Les fréquences exprimées en pourcentage et les moyennes avec leurs écart-types ont été calculés. Le test de Chi-carré et le test exact de Fisher lorsque recommandés ont été utilisés pour la comparaison des fréquences au seuil de signification de p<0,05.

**Résultats:**

La fréquence moyenne de césarienne était de 10,65%. Elle est passée de 10,24% en 2009 à 11,38% en 2013 soit une croissance de 11,13% (p=0,03). Cinquante-un virgule quatre pourcent de CS étaient effectuées après tentative de la voie basse et 48,6% dans un contexte d'urgence obstétricale. Les indications sont restées constantes et dominées par la disproportion fœto-pelvienne (18,6%), la souffrance fœtale aiguë (11,9%), la mal présentation fœtale (10,1%), le placenta prævia (9,2%) et le bassin rétréci (8,4%). La morbidité maternelle s'élève à 11,6% matérialisée par les complications hémorragiques dans 6,1%. La mortalité maternelle globale est passée de 2,3‰ en 2009 à 6,4‰ en 2013 soit une inflation de 178,26%. Pour la voie haute, elle est passée de 4‰ à 16,7‰ pour les 4 dernières années prises ensemble équivalant à une inflation de 317,5% (p=0,005) contre 2,1‰à 2,8‰ (33,33% d'inflation ; p=0,296) pour la voie basse au cours de la même période. La mortalité périnatale globale a connu une augmentation de 56,04% l'année 2010 (p=0,0000) et 43,36% en 2013 (p=0,0000). Concernant la CS, l'on notera une amélioration de la mortalité périnatale de 48,64% les trois premières années suivant 2009 (59,4‰contre 70,5‰; p=0,3278), amélioration à laquelle succédera une hausse de 46,52% (p=0,026). Globalement, la mortalité périnatale n'aura pas été significativement modifiée par la dynamique évolutive de la CS durant ces 5 années.

**Conclusion:**

Les cinq dernières années, la pratique de la CS à Lubumbashi (RDC) a connu une croissance de plus de 11% sans évolution de ses indications ni dividendes maternelles et périnatales.

**Introduction:**

The frequency of cesarean section (CS) has been steadily growing over recent decades but this growth varies enormously from one country to another and in the same environment as well as from one medical institution to another. This study aims to investigate the frequency, the indications and the maternal and perinatal morbi-mortality in patients undergoing cesarean section (CS) in Lubumbashi, Democratic Republic of Congo (DRC).

**Methods:**

We conducted a multicenter, retrospective, descriptive and analytic study of 3643 consecutive CS out of a total of 34199 deliveries in five hospital referral departments in Lubumbashi (DRC) between 1 January 2009 and 31 December 2013. We analyzed sociodemographic data,indications, obstetrical environment and perinatal morbi-mortality using Epi Info 2011 software. We calculated the frequencies expressed as a percentage and the means expressed in terms of standard deviations. We used chi-square test and Fisher exact probability test, when recommended, for the comparison of the frequencies having a significance threshold of p<0.05.

**Results:**

The mean frequency of CS was 10.65%. It rose from 10.24% in 2009 to 11.38% in 2013, corresponding to growth of 11,13% (p=0.03). 51.4% of CS were performed after attempting vaginal birth and 48.6% were performed in a context of obstetric emergency. Indications were constant and dominated by fetopelvic disproportion (18.6%), acute fetal distress (11.9%), fetal malpresentation (10.1%), placenta previa (9.2%) and narrowed basin (8.4%). Maternal morbidity rose to 11.6%, it was characterized by bleeding complications in 6.1%. Overall maternal mortality rose from 2.3‰ in 2009 to 6.4‰in 2013, corresponding to growth of 178,26%. Cesarean section rose from 4‰ to 16.7‰ in the last 4 years taken together, corresponding to growth of 317.5% (p=0.005) versus 2.1‰ to 2.8‰ (growth of 33.33%; p=0.296) for vaginal birth during the same period. Overall perinatal mortality rose to 56,04% in 2010 (p=0.0000) and 43.36% in 2013 (p=0.0000). With regard to CS, there was a reduction in perinatal mortality of 48,64% in the first three years after 2009 (59.4‰ versus 70.5‰; p=0,3278), this reduction was followed by an increase of 46,52% (p=0.026). Overall perinatal mortality wasn't significantly altered by changing dynamics in CS during these 5 years.

**Conclusion:**

In the last five years, CS in Lubumbashi (DRC) experienced growth of more than 11% without neither indications changes nor maternal and perinatal dividends.

## Introduction

La fréquence de la césarienne (CS) ne cesse d´augmenter ces dernières décennies mais cette augmentation diffère énormément d'un pays à un autre et dans un même milieu, d'une institution médicale à une autre [1, 2]. Les raisons sont multifactorielles et pas toujours défendables: les changements dans les caractéristiques maternelles et les styles de pratique professionnelle, l´augmentation de la pression en cas de faute professionnelle, ainsi que les facteurs économiques, organisationnels, sociaux et culturels ont tous été impliqués [3, 4]. A ceux-ci s'ajoutent les progrès dans la chirurgie, l´anesthésiologie et la transfusion sanguine assurant une certaine sécurité dans la réalisation de la césarienne [5]. Dans les pays à faibles ressources, la fréquence des césariennes est passée de 1,9% à 6,1% [1]. En République Démocratique du Congo (RDC), les dernières Enquêtes Démographiques et de Santé rapportent que les taux de CS sont passés de 4% en 2007 [6] à 5% en 2013 [7]. A mesure que la fréquence des césariennes augmente, l'importance relative des diverses indications change. Bien que les taux de césariennes électives soient de plus en plus élevés dans les pays développés [8], les césariennes d'urgence restent encore trop fréquemment pratiquées en Afrique subsaharienne [9, 10] malgré l'effet délétère sur le pronostic fœtal et maternel en augmentant significativement la morbidité et la mortalité y relatives [11]. Cette étude vise à déterminer la fréquence de la CS, ses indications, leurs évolutions et la morbi-mortalité maternelle et périnatale y afférentes entre 2009 et 2013 à Lubumbashi, en RDC.

## Méthodes

L'étude s'est déroulée dans cinq formations hospitalières de référence de la ville de Lubumbashi (hôpital Gécamines/Sud, Hôpital SNCC, Cliniques Universitaires, Centre médical Afia-Don Bosco et Hôpital Jason Sendwe) du 1^er^ janvier 2009 au 31 décembre 2013. Il s'agit d'une étude rétrospective, descriptive et analytique portant sur toutes les CS réalisées sur l'ensemble des accouchements qui ont eu lieu durant la période d'étude. Le recrutement a été exhaustif. Les registres d'accouchements, les partogrammes, les comptes-rendus opératoires, et les fiches de nouveau-nés en néonatologie ont constitué les sources d'information. Les paramètres sociodémographiques (âge maternel, parité, statut matrimonial), les indications de césariennes, l'environnement obstétrical (suivi des consultations prénatales, évacuation obstétricale, âge gestationnel, contenu utérin) et la morbi-mortalité maternelle et périnatale (complications et issue) ont été analysés au logiciel Epi Info 2011. Les fréquences exprimées en pourcentages et les moyennes avec leurs écart-types ont été calculées ; le test de Chi-carré et le test exact de Fisher lorsque recommandés ont été utilisés pour la comparaison des fréquences au seuil de signification de p<0,05. Ethiquement, avant la récolte de données, les autorisations respectives des Médecins Directeurs des cinq formations hospitalières concernées et des comités provinciaux d'éthique ont été obtenues après que nous ayons exposé les objectifs et la méthodologie de l'étude. Les données étaient collectées de manière anonyme. L'étude n'a pas présenté de bénéfice direct pour les participants à l'étude.

## Résultats

### Fréquences, caractéristiques sociodémographiques des opérées et indications

Du 1^er^ janvier 2009 au 31 décembre 2013, les maternités des cinq formations hospitalières ont enregistré un total de 34199 accouchements dont 3643 césariennes soit une fréquence relative globale de 10,65%, ainsi que 34980 naissances. La fréquence annuelle a varié entre 10,20%, taux le plus bas enregistré en 2011 et 11,38%, taux le plus élevé observé en 2013 avec un pic intermédiaire en 2010 (11,32%). Par rapport au taux de 2009 (10,24%), celui de 2013 (11,38%) présente une hausse de 11,13% (p=0,03) (**Tableau 1**). L'âge maternel moyen était de 28,83±6,79 ans (extrêmes : 14 à 49 ans). Sept opérées sur dix avaient un âge entre 20 et 35 ans. Les adolescentes (âge <20 ans) représentaient 10% des accouchées. La parité moyenne était de 2,6±2,5 (extrêmes : 0-16). Trois patientes sur dix étaient à leur premier accouchement et 92,1% s'étaient déclarées mariées. Dix virgule neuf pourcent des césarisées étaient porteuses d'un utérus cicatriciel de césarienne antérieure, 22,3% étaient des évacuées obstétricales et et 16% n'avaient pas suivi de consultations prénatales. S'agissant du terme de la grossesse, il a varié entre 22 et 47 SA, soit une moyenne de 37,9±2,8SA ; 5% des césarisées l'ont été sur des grossesses d'âge inférieur à 32 SA (Tableau 2). Un peu plus de 30% des indications justifient à elles seules 58,2% des césariennes, il s'agit (par ordre de grandeur décroissante) de la disproportion fœto-pelvienne (défaut d'engagement 18,6%), de la souffrance fœtale aiguë (11,9%), de la malprésentation (10,1%), du placenta prævia (9,2%) et du bassin rétréci (8,4%). La rupture et la pré-rupture utérines ont été enregistrées dans 4,9% des cas et la césarienne programmée dans 1,3% (Tableau 3). Les indications de césariennes étaient maternelles dans 35,9% des cas. Cinquante-un virgule quatre pourcent de césariennes étaient effectuées après tentative de la voie basse et 39,4% pratiquées dans un contexte d'urgence obstétricale (Tableau 4). L'analyse de la dynamique annuelle des indications opératoires montre qu'il n'y a pas eu apparition des nouvelles indications au cours de la période d'étude et celles enregistrées ont montré des taux stables et voisins du taux moyen à l'exception de la souffrance fœtale aigue et de la dystocie dynamique dont les taux dessinent des trajectoires en dents de scie avec des variations annuelles allant du simple au double (Tableau 3). Les causes maternelles représentent 39,7% des indications opératoires en 2013 contre 36% en 2011 et 32% en 2009 exprimant une augmentation annuelle de 6% alors que les indications fœtales sont passées de 26% en 2009 à 15,6% en 2013 signifiant une chute annuelle de 10%. Les indications annexielles ont connu une croissance globale de 32,8% alors que les mixtes ont globalement régressé de 5,38% (Tableau 4). La distribution des indications selon le caractère et le type par période montre que la césarienne était pratiquée en urgence dans deux cas sur cinq indépendamment de la période considérée (p=0,09) alors que 48,6% des indications revêtaient un caractère prophylactique (Tableau 4).

**Tableau 1:** Fréquence de la césarienne et son évolution

Année	Total Accouchements	Effectif VB	Effectif VH	Taux VH (%)	p
**2009**	7430	6669	761	10,24	0,038
**2010**	6541	5800	741	11,32	0,033
**2011**	7182	6449	733	10,20	0,983
**2012**	6607	5932	675	10,21	0,031
**2013**	6439	5706	733	11,38	
**Total**	**34199**	**30556**	**3643**	**10,65**	

VB : voie basse ; VH : voie haute

**Tableau 2:** Distribution des césarisées selon les caractéristiques sociodémographiques et cliniques

Variable	Effectif	Pourcentage
**Âge maternel (n=3475)**		
<20 ans	348	10,0
20-35 ans	2509	72,2
>35 ans	618	17,8
Moyenne (extrêmes)	28,8±6,8 ans (14-49 ans)	
**Parité (n=3439)**		
0	1042	30,3
1-2	968	28,1
3-4	627	18,2
≥5	802	23,3
Moyenne (extrêmes)	2,6±2,5 (0-16)	
**Statut matrimonial (n=3475)**		
Mariée	3202	92,1
Célibataire	273	7,9
**Utérus cicatriciel de césarienne (n=3537)**		
Non	3209	89,1
Oui	387	10,9
**Consultations prénatales (n=3333)**		
Suivies	2800	84,0
Non suivies	533	16,0
**Évacuation obstétricale (n=3491)**		
Non	2711	77,7
Oui	780	22,3
**Âge gestationnel (n=3346)**		
˂32,0 SA	163	4,9
32,0-36,6 SA	1016	30,4
≥37,0 SA	2167	64,7
**Contenu utérin (n=3643)**		
Monofoetal	3450	94,7
Gémellaire	184	5,1
Triplet	9	0,2

SA: semaines d’aménorrhée

**Tableau 3:** Distribution annuelle des indications de césarienne

Indications	2009		2010		2011		2012		2013		Total	
	n	%	n	%	n	%	n	%	n	%	n	%
Défaut d’engagement	122	17,9	96	15,0	113	17,5	109	17,4	178	24,3	618	18,6
SFA	92	13,5	100	15,6	68	10,5	85	13,6	50	6,8	395	11,9
Malprésentation	85	12,5	68	10,6	65	10,1	53	8,5	64	8,7	335	10,1
Placenta prævia	47	6,9	57	8,9	62	9,6	56	9,0	84	11,5	306	9,2
Bassin rétréci	54	7,9	43	6,7	69	10,7	52	8,3	62	8,5	280	8,4
Eclampsie	44	6,5	39	6,1	48	7,4	53	8,5	52	7,1	236	7,1
Pré-éclampsie	18	2,6	24	3,7	24	3,7	19	3,0	59	8,0	144	4,3
Travail prolongé	21	3,1	49	7,6	26	4,0	25	4,0	16	2,2	137	4,1
Utérus cicatriciel	23	3,4	26	4,0	21	3,3	36	5,8	22	3,0	128	3,8
DPPNI	29	4,3	22	3,4	26	4,0	17	2,7	23	3,1	117	3,5
Rupture utérine	22	3,2	15	2,3	22	3,4	24	3,8	21	2,9	104	3,1
Dystocie dynamique	14	2,1	10	1,6	12	1,9	22	3,5	45	6,1	103	3,1
Procidence	9	1,3	22	3,4	30	4,7	14	2,2	15	2,0	90	2,7
Pré-rupture	20	2,9	11	1,7	13	2,0	3	0,5	14	1,9	61	1,8
Césarienne programmée	15	2,2	7	1,1	1	0,2	10	1,6	11	1,5	44	1,3
Autres	65	9,6	53	8,3	45	7,0	47	7,5	17	2,3	227	6,8
Total	680	100	642	100	645	100	625	100	733	100	3325	100

SFA: souffrance fœtale aigue ; DPPNI: décollement prématuré du placenta normalement inséré

**Tableau 4:** Distribution annuelle des indications de césarienne selon le caractère, le type et l’étiologie

	Total (N=3325)	2009(n=680)	2010(n=642)	p*	2011(n=645)	p*	2012(n=625)	p*	2013(n=733)	p*
**Caractère**										
Urgent	1309 (39,4%)	263 (38,68%)	266(41,43%)	0,333	269 (41,71%)	0,285	252 (40,32%)	0,582	259 (35,33%)	0,000
Non urgent	2016 (60,6%)	417 (61,32%)	376(58,57%)		376 (58,29%)		373 (59,68%)		474 (64,67%)	
**Type**										
Électif	1617 (48,6%)	353 (51,91%)	304(47,35%)	0,109	324 (50,23%)	0,578	310 (49,60%)	0,435	326 (44,47%)	0,006
Non électif	1708 (51,4%)	327 (48,09%)	338(52,65%)		321 (49,77%)		315 (50,40%)		407 (55,53%)	
**Indication**										
Maternelle	1193 (35,9%)	216(31,8%)	217(33,8%)	0,092	235(36,4%)	0,000	234(36,4%)	0,079	291(39,7%)	0,000
Mixte	889 (26,7%)	202(20,5%)	156(24,3%)		159(24,7%)		166(26,6%)		206(28,1%)	
Fœtale	730 (22,0%)	177(26,0%)	168(26,2%)		133(20,6%)		138(22,1%)		114(15,6%)	
Annexielle	513 (15,4%)	85(12,5%)	101(15,7%)		118(18,3%)		87(13,9%)		122(16,6%)	

* valeur obtenue en comparaison du taux de l’année 2009; VH: voie haute; VB: voie basse

### Morbidité et mortalité maternelles et périnatales

La fréquence des complications per et postopératoires était de 11,6%. Celles-ci étaient dominées par l'hémorragie du post-partum rencontrée dans 52,77% des cas. La morbidité néonatale traduite par les différentes complications observées en période néonatale précoce s'élevait à 28,6% dont 78,2% des cas de détresse respiratoire (Tableau 5). Le ratio de la mortalité maternelle globale est passée de 230 décès maternels pour 100 000 naissances vivantes en 2009 à 640 décès maternels pour 100 000 naissances vivantes en 2013 (p=0,000) pour un ratio moyen de 390 décès maternels pour 100 000 naissances vivantes, ratio correspondant à un taux de mortalité maternelle (3,9‰) significativement supérieur à celui (2,3‰) de 2009 (p=0,036). La croissance de la mortalité maternelle pendant les 4 années succédant à 2009 est évaluée à 0,43% soit une augmentation de 86,96% pour une mortalité imputable à la voie haute (VH) passée de 4,0‰à 16,7‰ pour les 4 dernières années prises ensemble, ce qui équivaut à une augmentation de 317,5% (p=0,005) contre 2,1‰ à 2,8‰(soit une augmentation de 33,33% ; p=0,296), taux de mortalité imputables à la voie basse pour la même période (Tableau 6). Dans l'ensemble, l'on a répertorié 133 décès maternels sur un total de 34192 accouchements (3,9‰) toutes les voies confondues. La voie haute était concernée pour 51 décès sur 3636 opérations (14‰) contre 82 décès pour 30556 accouchements par voie basse (2,7‰) exprimant un risque létal multiplié par 5,29 pour l'accouchement par voie haute comparativement à la voie vaginale (OR=5,29 [3,67-7,61] ; p=0,0000). La mortalité périnatale globale est de 46,3‰ au cours de 5 années d'étude. Elle était à 33,9‰ en 2009 puis à 50,4‰, taux moyen des 4 dernières années soit une croissance de 16,50‰ correspondant à une hausse de 48,18% (p=0,000) alors que la part imputable à la VH était passée de 70,5‰ en 2009 à 70,8‰, taux moyen des 4 années ayant succédé à 2009 correspondant à une progression de 0,42% (p=0,981) contre une progression de 1,80% pour la mortalité par voie basse (p=0,000). En rapport avec la voie d'accouchement, la voie haute a été concernée pour 249 décès sur 3520 césariennes (70,7‰) contre 1349 sur 31017 accouchements par voie naturelle (43,5‰) signifiant un risque létal multiplié par 1,67 pour les nouveau-nés nés par voie haute (OR=1,67 [1,45-1,93] ; p=0,0000) (Tableau 6).

**Tableau 5:** Morbi-mortalité maternelle et périnatale

Variable	Effectif	Pourcentage
**Complications maternelles (n=3586)**		
Aucune complication	3171	88,4
Complications hémorragiques	219	6,1
Complications anesthésiques	56	1,6
Complications digestives et urinaires	43	1,2
Infections pariétales et endométriales	43	1,2
Infections respiratoires, affections cardio-vasculaires	8	0,2
Septicémie	6	0,2
Autres	40	1,1
**Évolution Maternelle (n=3636)**		
Survie	3585	98,6
Décès	51	1,4
**Complications néonatales (n=3377)**		
Aucune	2412	71,4
Détresse respiratoire	755	22,4
Traumatismes	54	1,6
Infections	33	1,0
Anémie	25	0,7
Autres	98	2,9
**Évolution périnatale (n=3520)**		
Survie	3271	92,9
Décès	249	7,1

**Tableau 6:** Mortalité maternelle et périnatale en rapport avec la voie d’accouchement

Mortalité maternelle	2009	2010	p*	2011	p*	2012	p*	2013	p*	Totaux
**Décès globaux**	7428 (100%)	6536 (100%)		7182 (100%)		6607 (100%)		6439 (100%)		34192 (100%)
Décès	17 (0,23%)	32 (0,49%)	0,009	25 (0,35%)	0,178	18 (0,27%)	0,605	41 (0,64%)	0,000	133 (0,39%)
Survie	7411(99,77%)	6504 (99,51%)		7157 (99,65%)		6589 (99,73%)		6398 (99,36%)		34059 (99,61%)
**Décès par VH**	759 (100%)	736 (100%)		733 (100%)		675 (100%)		733 (100%)		3636 (100%)
Décès	3 (0,40%)	16 (2,17%)	0,002	13 (1,77%)	0,011	5 (0,74%)	0,486	14 (1,91%)	0,006	51 (1,40%)
Survie	756 (99,60%)	720 (97,83%)		720 (98,23%)		670 (99,26%)		719 (98,09%)		3585 (98,60%)
**Mortalité périnatale**	**2009**	**2010**	p*	**2011**	p*	**2012**	p*	**2013**	p*	**Totaux**
**Décès globaux**	7591 (100%)	6445 (100%)		7315 (100%)		6365 (100%)		6466 (100%)		34537 (100%)
Décès	257 (3,39%)	341 (5,29%)	0,000	351 (4,80%)	0,000	335 (5,26%)	0,000	314 (4,86%)	0,000	1598 (4,63%)
Survie	7334(96,61%)	6104 (94,70%)		6964 (95,20%)		6030 (94,74%)		6152 (95,14%)		32939 (95,37%)
**Décès par VH**	723 (100%)	710 (100%)		736 (100%)		625 (100%)		726 (100%)		3520 (100%)
Décès	51 (7,05%)	48 (6,76%)	0,826	43 (5,84%)	0,345	32 (5,12%)	0,140	75 (10,33%)	0,026	249 (7,07%)
Survie	672 (92,95)	662 (93,24%)		693 (94,16%)		593 (94,88%)		651 (89,67%)		3271 (92,93%)

* valeur p obtenue en comparaison du taux de l’année 2009; VH: voie haute; VB: voie basse

## Discussion

### Fréquence

le taux de césariennes de 10,65% que nous rapportons dans notre série est enregistré en milieu hospitalier urbain et avoisine le double de ceux observés dans la population générale en République Démocratique du Congo [7]. En 2012, une étude menée dans 4 districts de santé de 3 pays africains (RDC, Burundi et Sierra Leone) trouvait un taux de césariennes moyen de 6,2% avec des extrêmes de 4,1% (à Masisi/RDC) et 16,8% (à Kabezi/Burundi) [9]. Une autre étude ayant analysé les données de 22 pays d'Afrique sub-saharienne montre que les taux d'accouchements par césarienne avaient augmenté au fil du temps dans chaque pays étudié, mais qu'ils étaient systématiquement inférieurs ou égaux à 10% voire inférieurs à 5% dans certains pays. Elle rapportait également qu'au Tchad, en Éthiopie, en Guinée, à Madagascar, au Mali, au Mozambique, au Niger et au Nigéria, les taux étaient restés en dessous de 1% dans la population des zones rurales [12]. Plusieurs facteurs justifieraient ces taux bas : la sous-qualification du personnel œuvrant au niveau des consultations prénatales [10, 13]; le manque d'accès à cet acte chirurgical car la plupart de médecins qualifiés vivent dans les zones urbaines et la plupart des hôpitaux présentent des infrastructures insuffisantes pour les soins chirurgicaux d'urgence [12, 14]; l'absence des moyens modernes de surveillance anté et intrapartale [10, 13] ; l'attitude ethnoculturelle pronataliste qui caractérise l'Afrique Sub-saharienne conférant à la césarienne un caractère trop limitatif de naissances, d'où la tendance à tout vouloir obtenir par voie naturelle [10, 13]. Par contre, certaines études africaines ont rapporté des taux supérieurs atteignant 20% ou plus: à Kinshasa (RDC), entre 2012 et 2013, les taux de césarienne ont varié de 28,5 à 31,2% [15, 16]; à Ouagadougou (Burkina Faso) un taux de 30,3% a été rapporté entre 2005 et 2008 [17], tandis qu'à Abidjan (Côte d'Ivoire) [18] et à Yaoundé (Cameroun) [11] des taux respectifs de 31,03 et 19,7% ont été notés. Il faut souligner qu'en Afrique Sub-saharienne où les taux de césariennes populationnels restent encore très bas, on assiste ces dernières années à une augmentation dans les hôpitaux de référence. De manière générale, le taux de césariennes est élevé dans les hôpitaux qui ont un score de complexité très élevé, dans les maternités privées ou appartenant à des organisations non gouvernementales et dans les établissements où il existe un système de motivation du personnel [19]. Selon l'OMS, les taux d'accouchements par césarienne au sein des établissements de soins varient considérablement en fonction de la composition des populations obstétricales qu'ils prennent en charge, de leurs capacités et de leurs ressources ainsi que de leurs protocoles de prise en charge clinique. En raison de ces différences, le taux de césariennes recommandé au niveau de la population ne peut pas être considéré comme le taux idéal au niveau de l'hôpital [20]. D'après les estimations les plus récentes, le taux global moyen mondial de césariennes est de 18,6%, allant de 6,0% à 27,2% respectivement dans les régions les moins et les plus développées. Les taux les plus bas se trouvent en Afrique (7,3%) et plus particulièrement en Afrique de l´Ouest (3%). Les taux les plus élevés se trouvent en Amérique latine et dans les Caraïbes (40,5%) et l´Amérique du Sud est la sous-région avec les taux moyens les plus élevés dans le monde (42,9%). Les pays avec les taux de césariennes les plus élevés dans chaque région sont le Brésil (55,6%) et la République dominicaine (56,4%) en Amérique latine et dans les Caraïbes, l´Egypte (51,8%) en Afrique, l'Iran et la Turquie en Asie (respectivement 47,9% et 47,5%), l'Italie (38,1%) en Europe, les États-Unis (32,8%) en Amérique du Nord et la Nouvelle-Zélande (33,4%) en Océanie [1]. In fine, quel serait le meilleur taux? N'est-t-il pas celui qui donne le meilleur résultat par rapport au bénéfice fœto-maternel: la réduction au moindre coût de la morbi-mortalité maternelle et périnatale lors de l'accouchement? Faisant donc dire qu'il ne doit pas exister un taux universellement consensuel étant donné que les problèmes obstétricaux sont différents d'un biotope à un autre.

### Evolution de la fréquence de la césarienne

le taux de césariennes annuel dans notre série a varié entre 10,20%, taux le plus bas enregistré en 2011 et 11,38%, taux le plus élevé observé en 2013, avec un pic intermédiaire en 2010 (11,32%). En rapport avec le taux initial (10,24% en 2009), le taux de l'année 2013 exprime un accroissement de 11,13% en 4 ans. Dans le même milieu, le taux de césariennes était de 1,4% en 1995 et 1,97% en 1999 selon une étude menée dans la plus grande formation hospitalière de la ville [10]. Nous pouvons donc conclure que le taux de césarienne a connu un accroissement de 478% en 14 ans et de plus de 677% en 18 ans. Au cours des dernières années, le taux de césariennes a connu une augmentation progressive dans toutes les régions du monde. Une récente étude ayant publié les données de 121 pays rapporte que, de 1990 à 2014, le taux moyen de césariennes était passé de 22,8 à 42,2% en Amérique Latine et aux Caraïbes, de 18,5 à 32,6% en Océanie, de 11,2 à 25% en Europe, de 22,3 à 32,3% en Amérique du Nord, de 4,4 à 19,5% en Asie et de 2,9 à 7,4% en Afrique. Durant cette période, l'Egypte est le pays qui a enregistré une hausse considérable de taux de césariennes dans le monde (de 4,6% à 51,8%) suivie de la Turquie, la Géorgie et la Chine [1]. Cette inflation de césariennes est survenue malgré les recommandations de l'OMS de ne pas dépasser 10 à 15% [21]. L'inflation des taux de césariennes pourrait s'expliquer notamment par : l'amélioration générale de la technique opératoire et de l'anesthésie- réanimation offrant plus de sécurité pour le couple mère-enfant [8, 22]; les progrès de la réanimation néonatale autorisant l'extraction des bébés hypotrophiques et prématurés avec une chance de survie sans séquelles neurologiques [23] ; le perfectionnement et, pour certains, l'utilisation excessive de la surveillance électronique de la parturition qui contribuerait à effectuer des césariennes parfois abusives [24, 25]; les changements démographiques intervenus dans les pays développés qui ont réduit le nombre moyen d'enfants par famille de sorte que chaque enfant devient plus précieux et que la limitation du nombre des naissances souvent imposée à tort ou à raison par la césarienne cesse d'être mal acceptée [10]; les connaissances obstétricales les plus récentes sur l'évolution de la mortalité et la morbidité maternelle liée à la césarienne élective ainsi que sur l'évolution de la mortalité et la morbidité maternelle et néonatale liée à l'épreuve du travail [22] ; le droit de la patiente de réclamer une césarienne élective si on reconnaît comme éthiquement indispensable, le devoir d'information et de consentement éclairé de la patiente sur les risques de l'épreuve du travail et de l'accouchement par voie basse [22]; les politiques de subvention des soins obstétricaux et néonataux d'urgence entre autres celles de gratuité de la césarienne mises en œuvre à l'échelle nationale dans certains pays comme le Mali, le Sénégal et le Ghana [26, 27]; mais également certains facteurs culturels : en Chine, par exemple, il existe une croyance selon laquelle le moment de naissance d'un enfant influencerait son avenir (chance et destin) [4]. En plus des facteurs évoqués, l'on ne perdra pas de vue l'augmentation des césariennes liée à l'augmentation de l'âge des parturientes, l'augmentation des grossesses gémellaires, la systématisation de la césarienne lors des présentations du siège, de l'obésité et des utérus cicatriciels sans oublier la variabilité des définitions des dystocies selon les auteurs et les pratiques conséquentes [**28**]. Enfin, nous pouvons ajouter les motivations commerciales, de complaisance et de convenance personnelle (du médecin qui programme une césarienne pour aller en vacances).

### Indications de la césarienne

l'étude révèle qu'une césarienne sur deux était pratiquée dans un contexte d'urgence obstétricale, la césarienne programmée ne représente que 1,3% des indications. La place occupée par les césariennes d'urgence dans cette étude est beaucoup moins importante que celle d'autres auteurs dans notre pays et en d'autres régions en développement qui rapportent des taux de césariennes d'urgence variant de 58 à 96% et évoquent les diverses raisons notamment :l'absence des vraies consultations prénatales pouvant sélectionner et orienter prophylactiquement les gestantes à haut risque vers les structures spécialisées ; la recrudescence des maternités périphériques de fortune et non valides ; la sous-qualification du personnel œuvrant dans les maternités périphériques, ignorant des contre-indications de la voie basse et ne référant les gestantes que très tardivement ; l'ethnoculture pronataliste qui caractérise nos populations y compris la plupart des professionnels de santé consacrant la voie basse comme seule maternité heureuse, la césarienne étant réputée à tort limitative des naissances ; la mauvaise répartition des centres de santé et les difficultés d'accès aux structures de référence ; et enfin et ce n'est pas le moindre, la pauvreté, l'analphabétisme qui caractérisent nos populations. Il s'agit des facteurs communs et caractéristiques des pays en développement [10, 11, 16–18]. La littérature rapporte que, dans 50 à 70% des cas, la césarienne est décidée du fait d'indications multiples ou associées, la décision d'hystérotomie étant prise sur un faisceau d'arguments qui, isolés, n'auraient pas rendu l'intervention nécessaire [28]. Actuellement, hormis quelques attitudes systématiques propres à certaines équipes, le taux de césariennes pour les principales indications est assez fixe : trois sièges sur quatre sont césarisés, un tiers des utérus cicatriciels est recésarisé, la souffrance fœtale représente un quart des indications, les dystocies dynamiques 5 à 10% des indications tandis que les disproportions fœto-pelviennes gardent des proportions variables [28]. Dans l'ensemble, la forte recrudescence du taux de césariennes notée aux deux dernières décennies s'est accompagnée d'une forte diversification de ses indications grâce aux progrès technologiques, à la meilleure connaissance de l'obstétrique, aux performances réalisées dans le domaine de l'anesthésie-réanimation et de la néonatologie ainsi qu'à l'évolution socio-culturelle des populations.

L'augmentation des indications de césariennes en cas d'hypertension artérielle répond aux recommandations d'un consensus qui veut qu'une césarienne soit indiquée si l'HTA est sévère ou instable à partir de la 34^ème^semaine d'aménorrhée. En effet à cette période, le fœtus court plus de risque de mourir du fait de l'HTA que de la prématurité [18]. S'agissant de l'infection à VIH, elle est une indication de césariennes électives pour la plupart des auteurs européens car elle permet de réduire le risque de transmission mère-enfant [29]. Dans notre milieu, nous optons pour l'accouchement par voie basse en l'absence de contre-indications obstétricales. Cependant, cet accouchement par voie basse se fait sous protocole antirétroviral et des précautions sont à prendre pendant cet accouchement pour protéger le bébé et le personnel. La présence d'une cicatrice de césarienne influence l'attitude obstétricale en l'absence de consensus amenant à des options arbitraires les unes et les autres dans lesquelles la témérité est confondue avec le courage et la peur avec la précaution et conduisant à pécher par défaut ou par excès et qu'au final personne ne va au ciel parce qu'ayant péché [30, 31]. Dans notre série, les indications sont demeurées constantes et caractérisées par la prédominance des urgences. Sur l'ensemble des indications répertoriées, cinq sont en tête : la disproportion fœto-pelvienne (18,6%), la souffrance fœtale aiguë (11,9%), la malprésentation fœtale (10,1%), le placenta prævia (9,2%) et le bassin rétréci (8,4%). Les études antérieures menées au Pérou, en Afrique et en Asie présentent une distribution identique révélant la prépondérance des indications opératoires liées aux dystocies mécaniques, malprésentations fœtales et aux accidents hémorragiques, et ce indépendamment des taux des césariennes fort différents d'un centre hospitalier à un autre dans un même milieu, l'inflation de la césarienne au fil des années ne se faisant essentiellement que sur la recrudescence de ces indications d'extrême urgence [9, 17, 32, 33]. Ce particularisme commun à la majorité des pays en développement est la juste expression de l'environnement chirurgical, socio-économique et ethnoculturel caractérisant les pays pauvres [34].

Notons cependant des divergences chez ces mêmes auteurs quant à la fréquence de la souffrance fœtale comme indication opératoire. En RDC, en milieu urbain, Tshibangu rapportait une fréquence de 23% aux Cliniques Universitaires de Kinshasa alors qu'à la même époque les fréquences relevées en milieu semi-urbain (Mbuji-Mayi) et rural (Boende) du même pays étaient respectivement de 1,5% et 0% [35]. Au centre hospitalo-universitaire d'Antananarivo (Madagascar), Andriamady trouvait une fréquence de 38,1% [36]. Récemment, Ouédraogo et Kaur rapportaient des fréquences respectives de 22,9% et 30,77% des césariennes indiquées pour une souffrance fœtale [17, 37]. Cette disparité de la fréquence des césariennes pratiquées pour une souffrance fœtale s'expliquerait notamment par les raisons suivantes: le taux des césariennes est généralement plus élevé dans les institutions universitaires que dans les hôpitaux généraux et la souffrance fœtale comme d'ailleurs d'autres dystocies sont susceptibles d'y être plus facilement et fréquemment diagnostiquées grâce au savoir des prestataires et à l'infrastructure disponible ; la méthodologie et les critères de définition et d'appréciation de la souffrance fœtale diffèrent énormément dans nos milieux où le seul point commun est la perturbation du rythme du cœur fœtal ; la souffrance fœtale comme diagnostic préopératoire pourrait être retenue comme diagnostic final au mépris de la dystocie initiale qui l'a générée (dystocie dynamique, dystocie mécanique, décollement prématuré du placenta normalement inséré,…), créant ainsi l'inflation d'une conséquence au détriment de la cause initiale. Notons également que la rupture et la pré-rupture utérines ont été enregistrées dans 4,9% des cas, taux hautement supérieur à ceux rapportés dans les pays développés et émergents [30, 38] mais voisins de celui de la plupart des régions subsahariennes et témoins de la qualité de surveillance intrapartale et du pouvoir éthnoculturel pronataliste sacralisant la voie vaginale comme seule véritablement heureuse même en plein milieu urbain [9, 10].

### Mortalité maternelle et périnatale

le ratio de mortalité maternelle et le taux de mortalité périnatale chez les accouchées et les nouveau-nés par voie haute sont respectivement de 1400 pour 100 000 naissances et 70,7 pour 1000 naissances contre 268 pour 100 000 naissances et 43,5 pour 1000 naissances chez les accouchées et nouveau-nés par voie naturelle au cours de la même période. Le risque de mortalité maternelle était statistiquement majoré chez les opérées comparativement à l'accouchement par voie naturelle (OR=5,29 [3,67-7,61] ). Gonzales et Esteves-Pereira, dans leurs études menées au Pérou et au Brésil, rapportaient que la césarienne était indexé d'un risque de décès maternel de 5,5 et 2,9 fois plus élevé comparativement à la voie basse [32, 39]. Il en est de même concernant la mortalité périnatale, le risque létal est très élevé chez les nouveau-nés par voie haute ; dans nos conditions de travail, cette voie apparait hautement non protectrice du bien être périnatal (OR=1,67 [1,45-1,93] ). Dans les pays développés, la recrudescence de la césarienne s'est accompagnée d'une baisse spectaculaire de la mortalité maternelle et périnatale [22]. Les faits observés dans cette étude, comme d'ailleurs dans la plupart des pays en développement, à savoir une prépondérance des indications d'urgence, une rareté des indications électives et une forte mortalité maternelle et périnatale se justifieraient non seulement par l'absence des moyens modernes de surveillance de la grossesse et de la parturition, le bas niveau socio-économique et intellectuel des populations, la carence en personnel qualifié dans les maternités périphériques, le sous-équipement qui caractérise nos institutions sanitaires, mais aussi et surtout par le rôle que joue encore l'ethnoculture dans les comportements de la reproduction même en plein milieu urbain [9, 10, 13]. Le culte de la maternité heureuse, que seule la voie naturelle symbolise, explique en partie la haute morbidité fœtale en période intrapartale et néonatale ainsi que la forte mortalité maternelle observées dans cette étude [35].

## Conclusion

Au cours des cinq années concernées par notre étude (de 2009 à 2013), la pratique de la CS a connu une croissance de plus de 11% sans évolution de ses indications ni dividendes maternelles et périnatales dans la ville de Lubumbashi en République Démocratique du Congo.

### Etat des connaissances actuelle sur le sujet

- La recrudescence de la césarienne dans les pays développés s'est accompagnée d'un bénéfice proportionnel pour le couple mère-enfant;
- En Afrique subsaharienne, la césarienne reste une opération qui s'accompagne d'une forte morbidité et mortalité maternelles et périnatales dans nos conditions de travail;
- Aucune donnée récente disponible sur ce sujet à Lubumbashi.

### Contribution de notre étude à la connaissance

- Première étude multicentrique dans notre contexte, à Lubumbashi, République Démocratique du Congo;
- L'étude proposée est la première étude analytique globale permettant de déterminer la fréquence de la césarienne, ses indications, leurs évolutions dans le temps;
- L'étude permet également d'évaluer le pronostic maternel et périnatal dans notre contexte.

## Conflits d’intérêts

Les auteurs ne déclarent aucun conflit d'intérêts.
